# Evaluating Muscle Synergies With EMG Data and Physics Simulation in the Neurorobotics Platform

**DOI:** 10.3389/fnbot.2022.856797

**Published:** 2022-07-12

**Authors:** Benedikt Feldotto, Cristian Soare, Alois Knoll, Piyanee Sriya, Sarah Astill, Marc de Kamps, Samit Chakrabarty

**Affiliations:** ^1^Robotics, Artificial Intelligence and Real-Time Systems, Technical University of Munich, Munich, Germany; ^2^School of Biomedical Sciences, Faculty of Biological Sciences, University of Leeds, Leeds, United Kingdom; ^3^School of Computing, University of Leeds, Leeds, United Kingdom; ^4^Leeds Institute for Data Analytics, Leeds, United Kingdom; ^5^The Alan Turing Institute, London, United Kingdom

**Keywords:** EMG, Neurorobotics, muscle synergies, biomechanics, simulation, muscle control, spinal cord

## Abstract

Although we can measure muscle activity and analyze their activation patterns, we understand little about how individual muscles affect the joint torque generated. It is known that they are controlled by circuits in the spinal cord, a system much less well-understood than the cortex. Knowing the contribution of the muscles toward a joint torque would improve our understanding of human limb control. We present a novel framework to examine the control of biomechanics using physics simulations informed by electromyography (EMG) data. These signals drive a virtual musculoskeletal model in the Neurorobotics Platform (NRP), which we then use to evaluate resulting joint torques. We use our framework to analyze raw EMG data collected during an isometric knee extension study to identify synergies that drive a musculoskeletal lower limb model. The resulting knee torques are used as a reference for genetic algorithms (GA) to generate new simulated activation patterns. On the platform the GA finds solutions that generate torques matching those observed. Possible solutions include synergies that are similar to those extracted from the human study. In addition, the GA finds activation patterns that are different from the biological ones while still producing the same knee torque. The NRP forms a highly modular integrated simulation platform allowing these *in silico* experiments. We argue that our framework allows for research of the neurobiomechanical control of muscles during tasks, which would otherwise not be possible.

## 1. Introduction

We take our freedom to move around at will for granted. Only when this freedom is impaired do we start to realize how much so, and only when we try to alleviate the impairment with the aid of technology do we start to fully appreciate how complex the pathway from intended motion to execution actually is. Much of our normal walking is unconscious, driven by rhythmic neural activation generated in the spinal cord by the central pattern generators. It has long been obvious that the spinal cord has considerable autonomy in calculating appropriate responses to proprioceptive input and that cortical intervention would be too slow.

It is still unclear though how precise motor output is generated based on the integration and balancing of cortical input and proprioceptive feedback. The neural circuitry of the spinal cord has not been mapped functionally at the same level of detail as that of the cortex. This is because much of the knowledge gained through invasive techniques, e.g., multi-electrode arrays, cannot be performed in humans, except in unusual circumstances. The generalization of ideas that were developed in models of four-legged creatures to human performance is clearly problematic.

A non-invasive technique is electromyography (EMG), where relatively small wearable sensors are used to record muscle activity. In this work we use the data from an experimental study of an isometric human knee extension where the role of feedback in recruitment of muscles for the act, was established (York et al., 2022). As per standardized and well-established protocols, muscle activation is recorded from five distinct muscles. Together with neural modeling, such measurements can provide insight in local processing in the spinal cord. But importantly, even in animal studies there is still no access to individual muscle forces.

Simulation is the only available option to understand how force is generated once the activation pattern has been established. The Neurorobotics Platform (NRP) (Knoll and Gewaltig, 2016; Hinkel et al., 2017; Albanese et al., 2018) developed within the Human Brain Project (Markram et al., 2011; Amunts et al., 2016) is a natural simulation environment for gaining an understanding of force vector output by neural signals, as we can model the entire system: neural activation, resulting muscle tensions and resulting force output, in this experiment the resultant force on the knee torque. In this paper, we extend the feature rich framework of the NRP for EMG data evaluation and demonstrate that it is ideally suited to investigate force generation in a musculo-skeletal model. Moreover, since it is an entirely simulated environment, this allows *in silico* experimentation. We apply machine learning and signal processing to reanalyze the EMG data trials, determine features and cover the synergies in the signal. We feed the muscle activation into the physics simulation, observe resulting biomechanical motions and quantify resulting knee torques.

We use a Genetic Algorithm (GA) to generate alternative control signals as a reference that produce the same torque output as in the study. Musculoskeletal control systems are highly redundant, therefore calculating the inverse dynamic solution remains to be difficult and we instead use a bio-inspired approach based on heuristics search. GA is a parallel algorithm with a multitude of different initial populations that cover a wide search space, in our case a multitude of muscle activation configurations, and hereby is able to find alternative synergies that produce the same torque on the simulated model. Preserving multiple potential solutions throughout the algorithm execution is a benefit to foster diverse solutions in contrast to, e.g., gradient based methods. GA are a natural fit in use with a simulation environment since the fitness of any offspring can be evaluated online in a closed-loop of applying muscle activation to the simulated musculoskeletal model and observing generated joint torques. We choose GA over other bio-inspired methods such as Particle Swarm Optimization since its implementation does not require any global memory that, e.g., keeps track of the best solution of all execution steps. Hereby we lay the foundations for highly efficient search with parallel simulations of more complex control systems in the future, as the NRP provides a scripting interface to a high number of simulation instances that are running on virtual machines of a large computing center.

We have experimental evidence that demonstrates so-called *synergies* (York et al., 2022), the co-activation of several muscle groups to produce a desired motor output. With this work we can embed these findings in a larger context. We find that the same body behavior can be generated by various possible synergies, a number of the synergies found in our simulation matched those identified in the experimental data. This suggests that there is more than one way to produce a given motor output and we show that a simulated environment can be used to infer potential implementations for control strategies. Modeling helps understanding the considerable amount of redundancy that is available in the musculo-skeletal system and provides new insights in how the desired action may be executed.

## 2. Related Work

### 2.1. EMG Based Simulation

Apart from EMG recordings, the observability of muscle control principles in human is limited and therefore simulations based on EMG data are a central aspect with an increasing research interest for the study of body motion control. Research includes the simulation of physiology and morphology of muscles, e.g., models to generate EMG data (Schnetzer et al., 2001), or to compare it with biological recorded data (Hamilton-Wright and Stashuk, 2005).

Such EMG based simulations can enhance rehabilitation procedures and control of neuroprosthetic devices. Aung and Al-Jumaily (2011) proposes a real time visualization of colorized muscles based on EMG input to increase motivation in rehabilitation exercises. In Amezquita-Garcia et al. (2022), a musculoskeletal simulation has been used to visualize finger motions that are extracted from EMG data. In Blana et al. (2016), neural networks have been applied to compute arm joint positions from EMG data and resulting motions are visualized on an arm model in Virtual Reality for improved neuroprosthetic control. A musculoskeletal lower body model is simulated with OpenSim (Delp et al., 2007; Seth et al., 2018) and controlled by data from wearable EMG and IMU sensors in Cimolato et al. (2020), the predicted joint torque in simulation is used to control the subject's neuroprosthetic leg.

The combination of EMG data as a basis for activation control of virtual muscles and corresponding body motion data enables novel methods for the analysis of musculoskeletal control in simulation. Data from wearable EMG sensors has been applied in simulation for gait analysis in Gurchiek et al. (2022). Jonkers et al. (2002) showed that gait simulation based on EMG and joint data reflects kinesiological principles of walking even with a simplified musculoskeletal leg model and can serve as a basis for studying motion execution resulting from muscle control. A benchmark between different musculoskeletal simulators, here SIMM-SD/Fast and Opensim, with an upper limb model and EMG driven muscle activation can be found in Saul et al. (2015).

In Żuk et al. (2018), a musculoskeletal lower limb model, similar to the one we use in this work, has been simulated in OpenSim and muscles are controlled by EMG data to study the contribution of individual muscles to the final gait pattern, but no definite answer could be found. With our work we contribute another aspect to the research question of muscle activation patterns with a similar procedure, but demonstrate alternative muscle control strategies in an isolated knee bending experiment compared to EMG based muscle activation. We integrate the simulated body model in a 3D environment setting that recreates an experiment setup virtually, and the NRP supports the design of such musculoskeletal models and worlds with dedicated design tools (Feldotto et al., 2022). The implementation of spiking neural networks for muscle activation enables a comparison of muscle control paradigms with activation generated by simulated spiking neural network models of the spinal cord in the future.

### 2.2. Neurorobotics Platform

The Neurorobotics Platform (NRP) is developed within the European Human Brain Project and is integrated into the EBRAINS research infrastructure for brain simulation on supercomputing infrastructure. The NRP uses the robot simulator Gazebo (Koenig and Howard, 2004) for rigid body computations and has been extended with the muscle simulation framework from OpenSim (Delp et al., 2007; Seth et al., 2018) to simulate musculoskeletal models interacting with virtual environments. This unique integration enables simulation of classical motor-driven robots and biologically derived musculoskeletal mammalian bodies in the same experiment, in our case a musculoskeletal lower limb model in a virtual test environment. As part of the NRP the Closed Loop Engine is implemented to synchronize spiking neural network simulations in NEST (Gewaltig and Diesmann, 2007) with the physics simulation in predefined simulation steps. Transfer Functions specify the bidirectional exchange of sensory data and motor control commands. To optimize the workflow for experiment design the NRP provides user tools for fully customized setups such as a Robot Designer, Environment Designer, a Virtual Coach for scripted experiments and State Machine for automated control. Communication is based on the widely used Robot Operating System (ROS) (Quigley et al., 2009) that enables modular interchange of information within the NRP, as well as with external processing nodes in soft- and hardware. In this work we add an additional processing node within this modular framework for EMG data processing and control with a Genetic Algorithm. The NRP is deployed on the EBRAINS computing cluster infrastructure and hereby accessible *via* web browser. We here employ a local installation of the NRP on a local computer as it is offered open source to a wide user community. Use cases include the evaluation of neural networks based on neurophysiological experiments in embodied closed-loop simulations with musculoskeletal and robotic models.

The NRP has been used for reconstruction of physical experiments before, in particular a stroke rehabilitation study with mice has been implemented in Mascaro et al. (2020). Here, we focus on the human musculoskeletal system and EMG data of muscle activation that help us better understand biological muscular motion control patterns.

## 3. Experimental Setup

In this section, we describe the experimental study that has been realized in this work. We first introduce the participant study of human knee extension and then we describe the simulation architecture that has been implemented to reproduce and evaluate the study data virtually.

### 3.1. Human Knee Extension Study

We conducted a modified knee extension study with human participants (York et al., 2022) and focus on the knee angle alone as the one degree of freedom (DOF) at the rotary joint. All others will be avoided by providing support and one DOF also provided better measurable one dimensional feedback, reducing the effect of extraneous variables.

A cross-sectional, single-blinded control study was designed to compare the muscle activity at four distinct knee angles during isometric contractions. Healthy participants (*n* = 17, female = 8) within the ages of 18–30 (24.4 ± 2.57 years) without previous knee joint injury participated in the study. Participants were randomly allocated to different groups, and all measurements were conducted in the motor control laboratories of the center for sports sciences, in the School of Biomedical Sciences, University of Leeds, UK.

A physio-clear plastic Goniometer angle ruler was used to measure the knee extension angles. Participants lay supine on the bed with the head, back and leg muscles being fully supported. The setup is adapted from the Fugel-Meyer's knee control test used in stroke rehabilitation (Fugl-Meyer et al., 1975). The knee was held in position using a locking knee brace (DonjoyTM).

#### 3.1.1. Study Procedure

Subjects were asked to perform an isometric knee extension experiment with maximal voluntary effort. Initially, participants lay suspine on the bed with both legs stretched out in front, the hip, knee and ankle are located at a 0° to each other. For every recording the participant contracted their muscles at the back of the thigh of the right leg, while the knee is placed at a given steady angle. Participants were asked to activate the Rectus Femoris (RF) voluntarily, to ensure RF was the most active muscle, in line with current literature. We examined four different knee angles: at 0° (foot extended, so straight at the knee), 20° (knee is slightly bent with foot pointing away), 60° (middle of the range of knee flexion), and 90° (foot is at a perpendicular to the hip regarding the knee).

The order in which the knee angles were presented for testing was randomized across participants. Overall, participants contracted their muscles at each angle 6 times, with each contraction lasting 5 s, with a 3-min break between each contraction. We use anonymized indices consisting of three letters for data recorded from the various study participants such as *aac* and *aad*.

#### 3.1.2. EMG Data Recording

For all the four fixed knee angles with maximal muscle effort EMG data was recorded. For this purpose sensor pads were attached to record from the knee muscles Rectus femoris (RF), Vastus Lateralis (VL), Vastus Medialis (VM), Semitendinosus (Se), and Biceps Femoris (BF). For recording wireless sEMG sensors (Delsys TrignoTM system; at 1.9 kHz, the bandwidth of 20 to 450 Hz) were used. Electrode placement and recording sites were selected based on the SENIAM protocol (Rainoldi et al., 2004). We used anatomical landmarks to identify the muscles following the SENIAM guidelines (Rainoldi et al., 2004). Prior to placement of sEMG electrodes, relevant skin areas were shaved and cleaned with isopropyl alcohol, abraded with preparation gel (Nuprep, NRSign Inc., Canada), (Merletti et al., 1998). The electrode placement was verified in each subject by palpating the muscles and asking participants to perform a muscle contraction. The root-mean-square (RMS) of the evoked muscle activity was then calculated. Activity across muscles was normalized to that in RF, which was voluntarily activated by the participants at each angle in both tasks.

### 3.2. Simulation Architecture

**Figure 1:**
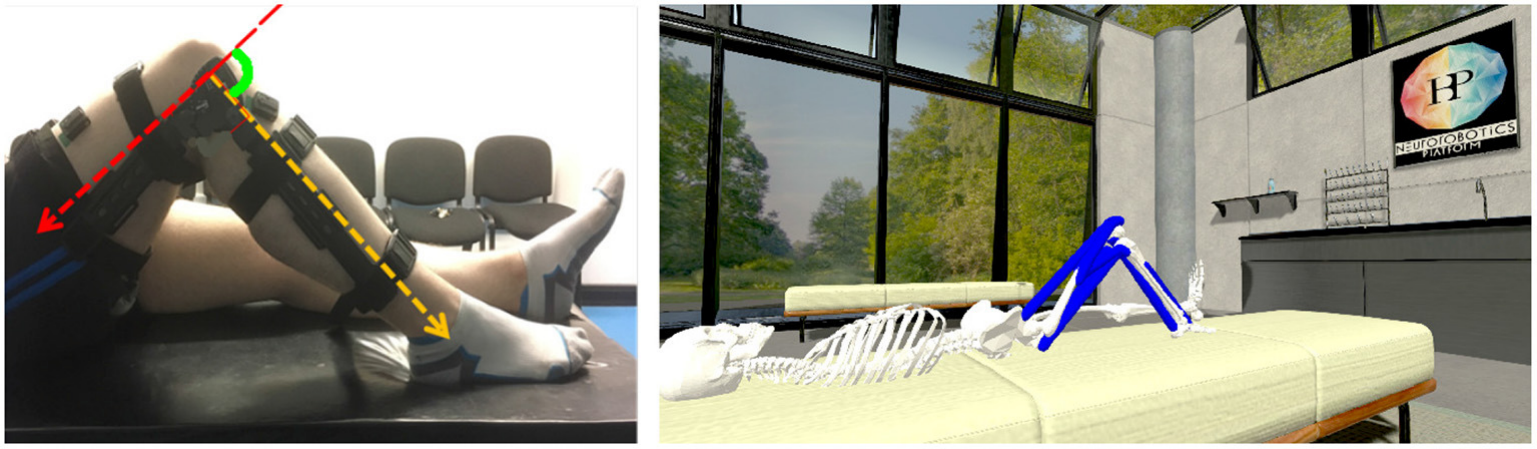
Human knee extension experiment: Participant study with EMG recordings **(left)** and experiment replication in the Neurorobotics Platform **(right)**.

EMG data analysis reveals insights into correlation between muscle activation and knee angle, but solely looking at the recorded EMG data a conclusion about resulting joint torques cannot be derived. We therefore reconstruct the physical human knee extension study in a simulated environment: We model a virtual room that contains an experiment bed and a musculoskeletal body model as a physics simulation in the NRP. Figure 1 shows both an exemplary test setup of the original participant study (left) and its replication with the musculoskeletal simulation as the basis for the experiments described in this paper.

**Figure 2:**
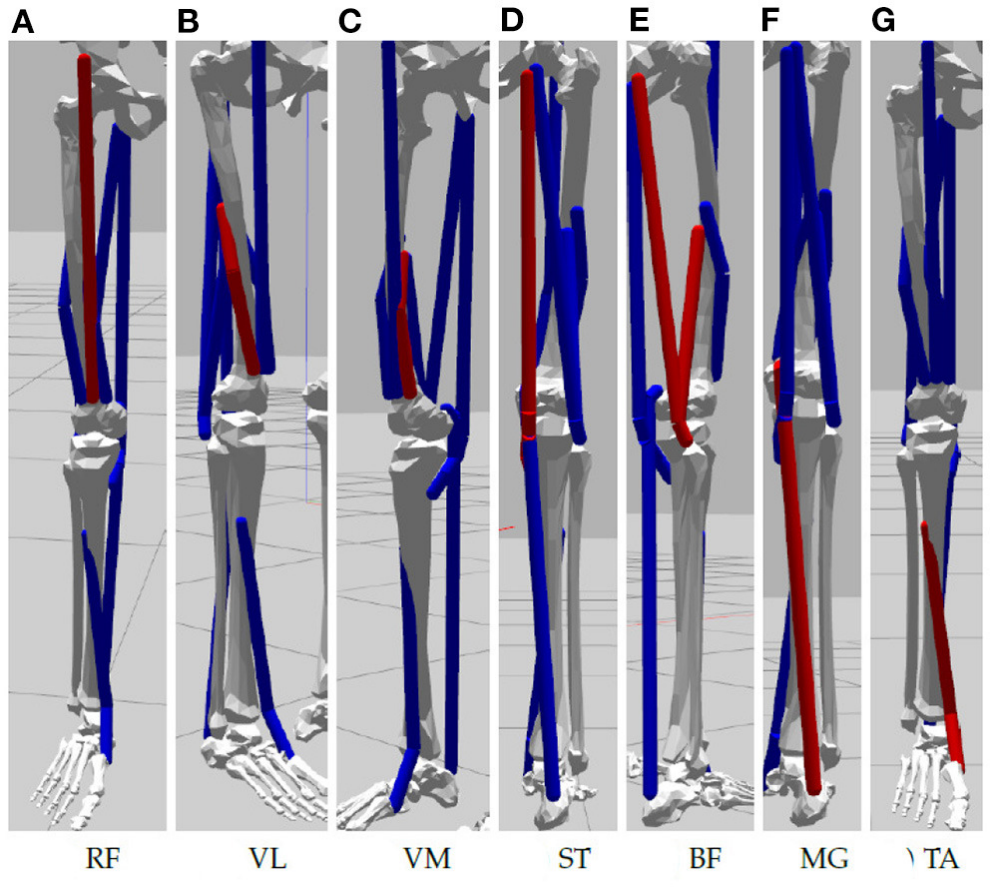
Muscles of the simulated lower limbs considered in our experiment: **(A)** Rectus Femoris, **(B)** Vastus Lateralis, **(C)** Vastus Medialis, **(D)** Semitendinosus, **(E)** Biceps Femoris, **(F)** Medial Gastrocnemius, and **(G)** Tibialis Anterior.

An available OpenSim model of the lower limb (Delp et al., 1990; Anderson and Pandy, 1999; Au and Dunne, 2013) is adapted for this study and its implementation in the NRP. The original model represents a subject that is 1.8 m tall and has a mass of 75.16 kg (Au and Dunne, 2013), mass and inertial properties have been averaged from anthropometric data of five subjects (age 26 ± 3 years, height 177 ± 3 cm, and weight 70.1 ± 7.8 kg) (Au and Dunne, 2013). To achieve best accuracy of musculotendon actuator paths bone surfaces had been marked with polygon meshes and then digitized (Delp et al., 1990). For our simulation study in the NRP joints are reduced to basic revolute motions and only muscles recorded in the physical study are used. These are visualized in Figure 2: Rectus Femoris (RF), Vastus Lateralis (VL), Vastus Medialis (VM), Semitendinosus (Se), Biceps Femoris (BF) as well Medial Gastrocnemius (MG) and Tibialis Anterior (TA). All muscles are simulated according to Thelen (2003), hill type models that emulate characteristics of elasticity and damping. The torso is fixated to the virtual bed, the right leg can move in a planar space in line with the physical setup. We implement a ROS control node in Python that controls simulated muscles with an activation value in range [0,1] (0 represents a fully relaxed muscle and 1 expresses maximal activation). Muscle synergies as well as resulting biomechanic behaviors measured in terms of joint angles and torques are recorded for analysis.

**Figure 3:**
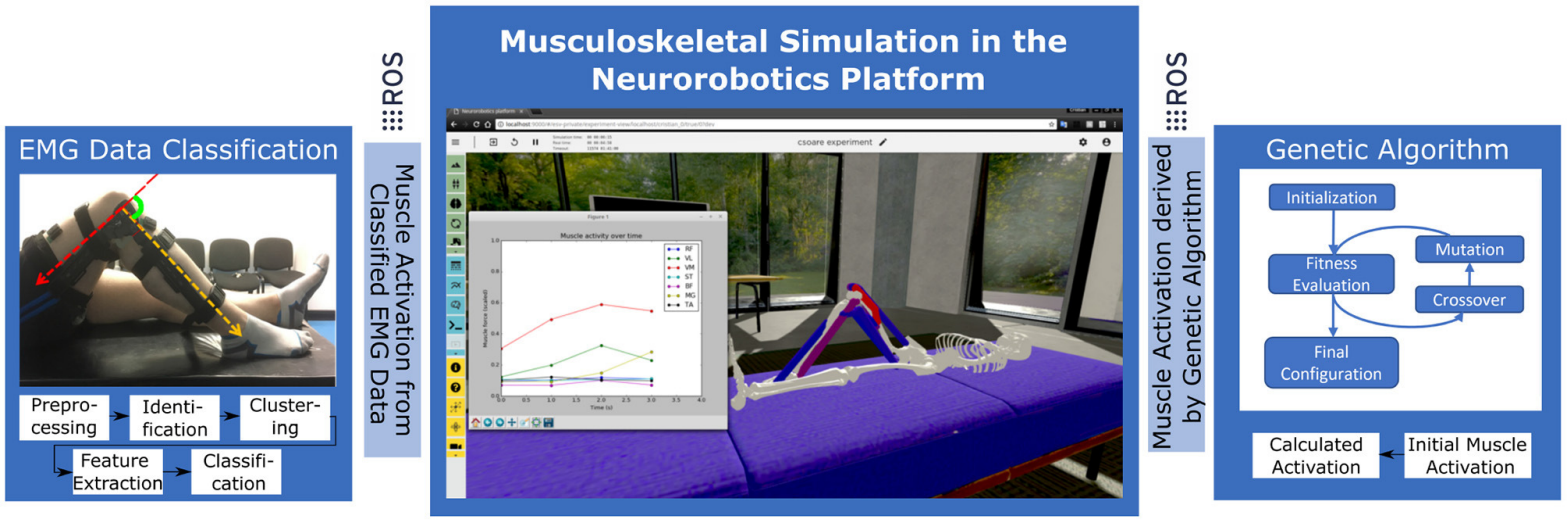
Experiment setup in the Neurorobotics Platform with two distinct control approaches: EMG data from the participant study is processed and applied to the simulated model to record resulting knee forces. In a second step optimal synergies are calculated by Genetic Algorithms using the simulation model as test and evaluation bed.

In this paper we demonstrate a pipeline that consists of two different control and evaluation steps to actuate the musculoskeletal model. First, we calculate normalized muscle activation values from EMG data collected during the participant study. Second, we implement GA to compute an optimal muscle control pattern by active trial on the simulated model. Figure 3 visualizes the architecture, that is centered around the virtual experiment replicate, conceptually. In both control steps we interface derived muscle activation data with the physics simulation by means of ROS topics. As a result we inspect and record knee joint angles and torques on the simulated model.

## 4. Data Processsing

Data passes through several steps before the final virtual experiment can be performed. First, the raw EMG data is preprocessed and muscle activations are extracted from the trial recordings. The normalized inputs are then fed into the NRP simulated muscles and the resulting torques are measured. Finally, these torques are taken as reference and a GA is used to generate muscle activation configurations which replicate the measured values.

### 4.1. EMG Data Analysis

#### 4.1.1. Trial Localization

**Figure 4:**
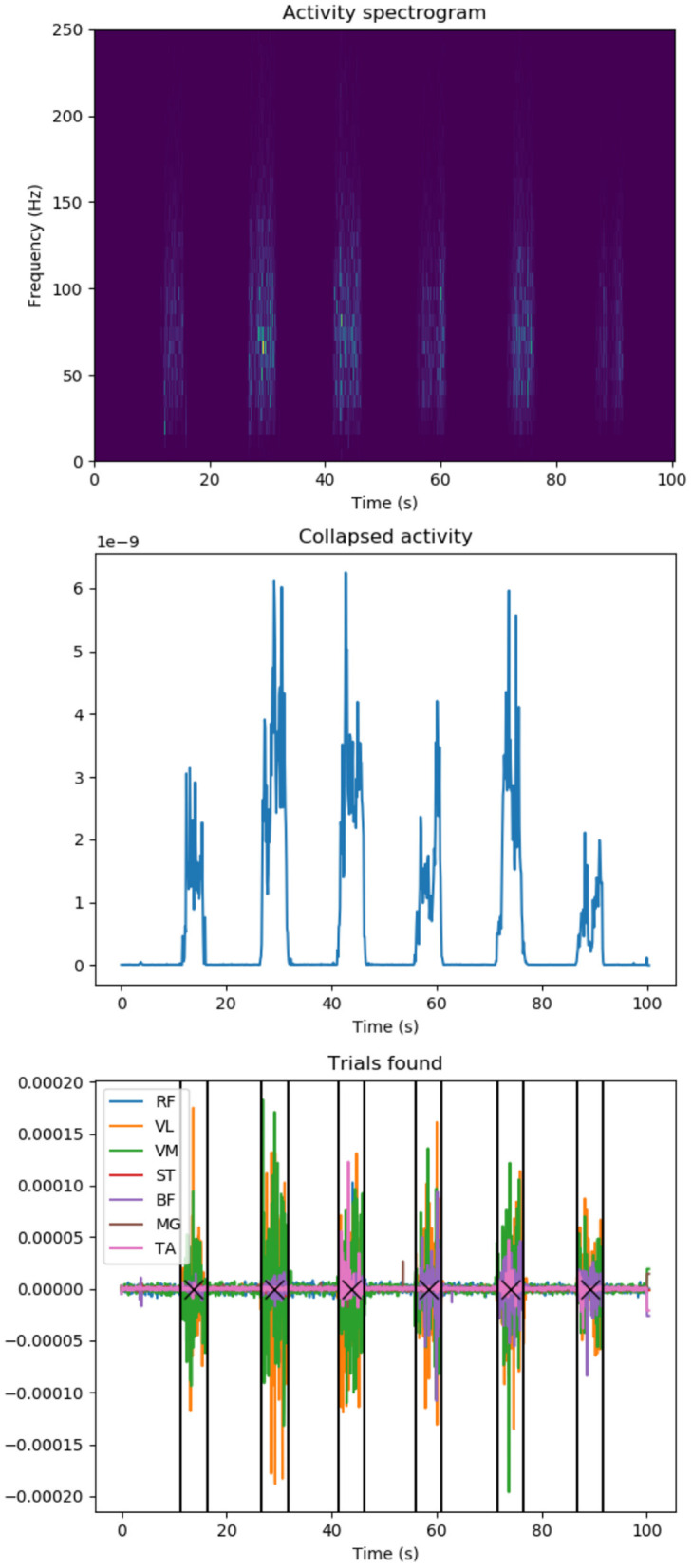
EMG data knee extension trial localization: **(top)** Activity spectrogram of all muscle recordings: Frequency spectograms for all recorded muscles are added up to identify overall muscle activation during a participant trial session. **(Middle)** Summed activity spectrogram: Frequencies are added up at any given time point to spot knee extension trial durations. **(Bottom)** Identified knee extension trials in original recorded EMG data (colors represent individual muscles): a participant executes several trials of knee extension in a row, we apply signal processing to identify trial duration (vertical lines) and centers (crosses). Y-axes values are in arbitrary units unless stated.

To perform the experiment, the trials have to be isolated and extracted from the original recordings and the EMG signals have to be preprocessed and readied for the simulation. As previously stated in Section 3.1.1, the experiments consists of 6 trials, each lasting 5 s, with a 3 min break in between. Signals are first transferred into frequency domain, rendering 7 spectrograms per experiment, corresponding to the 7 muscles of interest. The spectrograms are then summed into a single muscle activity spectrogram (Figure 4, top) which, in turn, is collapsed into a 2D plot (Figure 4, middle) by further summing the amplitudes over all frequencies at any given time point. Each time the activity plot is greater than a threshold determined by the SNR, an activity event is marked in time domain. Since it is known that each experiment has 6 trials, the K-means algorithm is used to find the trial origin of each activity event by clustering them in 6 clusters. Finally, the median time point of a trial's activity events determine its central time-wise occurrence (Figure 4, bottom). Since each trial is executed for 5 s according to experiment instructions, we identify each trial's onset and offset as its origin ±2.5 s.

#### 4.1.2. Signal Preprocessing

**Figure 5:**
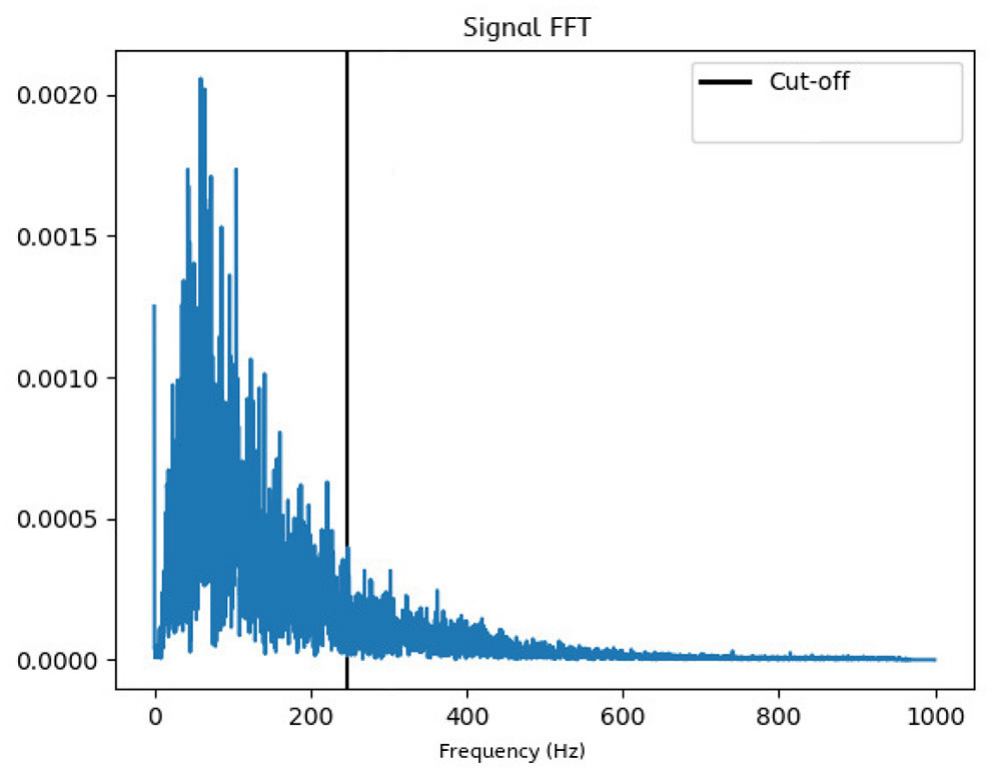
Frequency domain plot: The FFT-generated data is averaged over all trials, which shows that the most prominent frequencies lie within the lower end of the spectrum. All frequencies higher than the computed cut-off point will be ignored in further analysis.

The data contains noise components which have to be filtered out. Moreover, only a specific bandwidth may offer relevant information for further analysis. By observing the signals in frequency domain, it becomes evident that most high amplitude activity occurs in the lower end of the spectrum, as seen in Figure 5. Therefore, to increase the signal analysis reliability, we need to find a heuristic that automates calculating a cut-off point that is tailored to our data set. The FFT analysis showed that lower frequencies are of higher amplitude and are in fact a minority when compared the rest of the spectrum, a property which has proven useful in analyzing the signal from a statistical angle. The proposed heuristic is as follows: After averaging the FFT values across all study experiments and creating an amplitude-wise histogram of the frequencies, we fit a binomial distribution envelope to said histogram. We then find that the first outlier outside the standard deviation acts as a good amplitude threshold. The highest frequency reaching this threshold will therefore be considered the cut-off point.

#### 4.1.3. Feature Extraction

To be consistent with the study experiment, muscle activation data has to be inferred from the EMG signals. It is known that a muscle unit (MU) exerts a muscle unit activation potential (MUAP) when activated. An increased firing rate of a neuron translates into a higher stimulation of the muscle fibers, resulting in a sustained force (tetanic contraction) in the MU. Furthermore, due to the size principle in MU recruitment (Mendell, 2005), larger neurons, associated with a higher firing threshold are activated with the increase of force, which can be observed as an increase of voltage in the EMG recordings. Taking these into account, our heuristic to approximate muscle forces *F* equates to


$$F=\overset{high}{\underset{f=low}{\sum }}f\times v\left(f\right)$$

A weighted sum where each constituent frequency component *f* is multiplied by its amplitude *v*(*f*).

### 4.2. From Muscle Activation to Simulation Control

**Figure 6:**
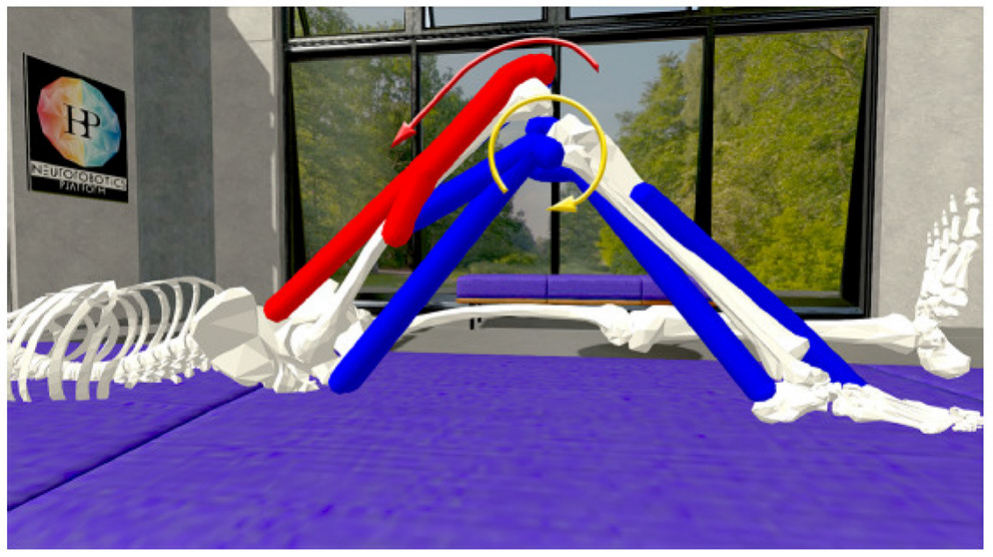
Knee torque evaluation on the simulated musculoskeletal model in the Neurorobotics Platform: Muscles are activated (muscle color coding: blue—no activation, red—maximal activation) by either processed EMG signals or a Genetic Algorithm and results in a muscle generated knee torque (red arrow). A counter acting knee torque (yellow arrow) is iteratively increased until the goal knee angle is reached.

Once the EMG recordings have been converted to muscle activation scalars, these values are normalized and applied on the NRP leg model muscles. Consequentially, the virtual muscles are contracted and the leg is extended with a resulting knee torque. The NRP does not provide a way to directly measure a muscle generated torque in a given joint. However, it does allow dynamic interaction with the model *via* its Python API. Therefore, by applying an increasingly stronger virtual torque opposing the muscle generated one (shown in Figure 6), once the leg reaches its original bent position it can be inferred that the artificial force equals the muscle driven one. The approach of applying torques that are increasing is supported by Beltman et al. (2004).

### 4.3. Genetic Algorithm to Compute Optimal Muscle Activation

Having extracted the knee torques from the original EMG recordings, using the NRP simulation, we can test whether the same rotational force can be achieved through different synergies. To do this, a GA is used in conjunction with the simulation to evolve an activation pattern that matches the target torque. The GA makes constant use of the simulation to measure the fitness of its population. In this problem context, each individual has 7 genes representing the activations for the controlled muscles. The initial individual is generated randomly. In every generation the fitness of each individual is defined by *cos* (|*a*_*current*_−*a*_*target*_|), where *a*_*current*_ is the knee angle observed in the simulation using the current individual's activations and *a*_*target*_ is the target angle of the algorithm. We implemented the GA based on the DEAP Python library (Fortin et al., 2012), the selected operators and parameters can be found in Table 1.

**Table 1:** Genetic Algorithm operators and parameters.

**Operation:**	**Crossover**	**Mutation**	**Selection**
Type:	cxTwoPoint	mutFlipBit	selTournament
Parameter:	Crossover probability = 0.5	Mutation probability = 0.2	Tournament size = 3
		Independent probability = 0.05	

*The Genetic Algorithm executed the three steps of Crossover, Mutation, and Selection iteratively until the predefined fitness is reached*.

While this evolutionary approach can find muscle configurations that match the target study experiment torques, it is not guaranteed that any given solution has at least a fully tensed muscle. Each gene within an individual is created randomly with a value between 0 and 1. This means that the configuration does not represent maximal voluntary effort. To mitigate this, the genes of an individual are scaled to the maximal value which becomes 1, while the others increase proportionally.

## 5. Results

### 5.1. EMG Data Analysis

**Figure 7:**
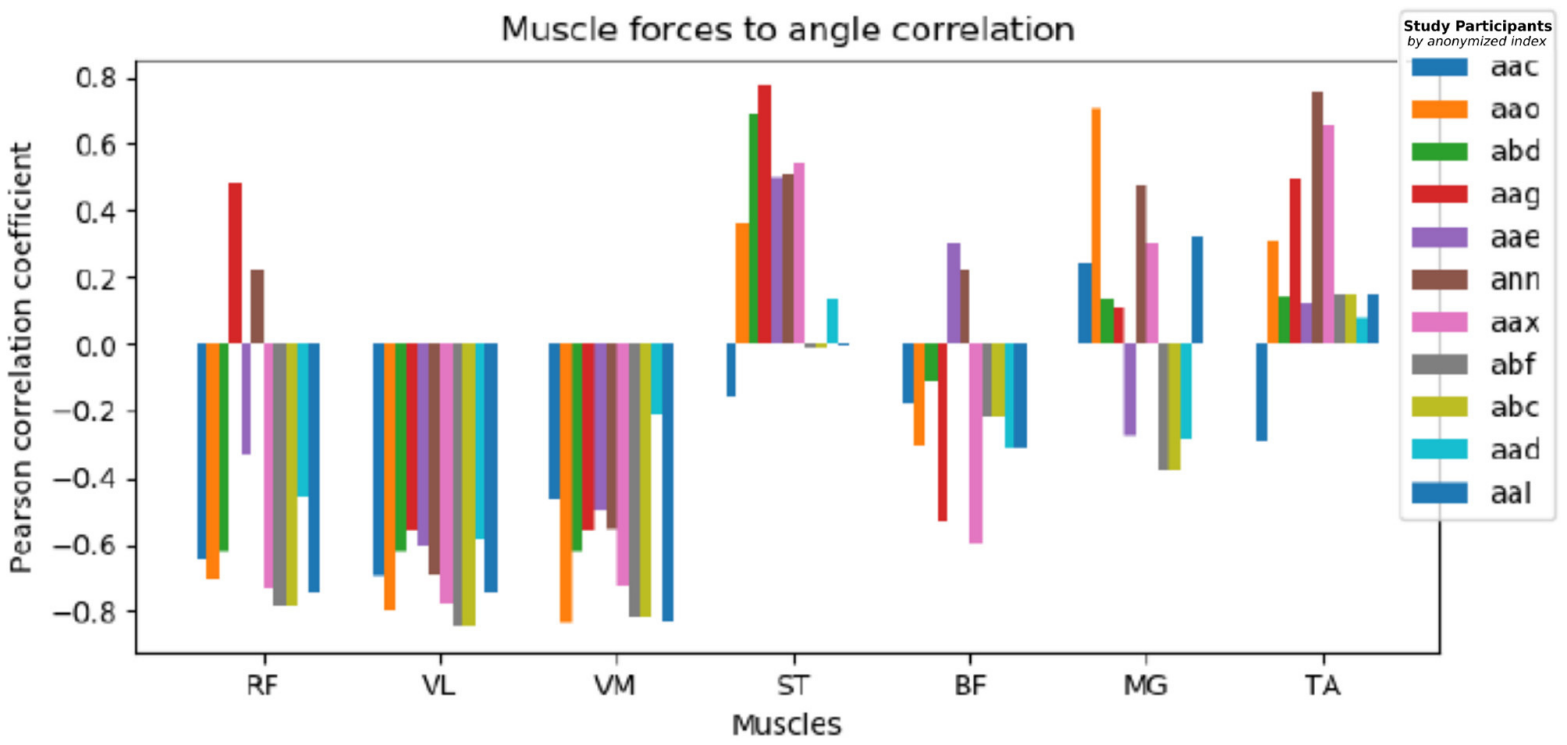
Muscle to knee angle Pearson correlations for multiple patients: Different colors refer to data of different study participants, the Pearson correlation is shown clustered by examined muscle groups. We observe a strong negative muscle activation correlation with the given angle for RF, VL, and VM, contrary ST and TA show a weak positive correlation and no definite correlation can be found common for all participants for BF and MG.

The muscle activation approximation heuristic described in Section 4.1.3 demonstrates results in accordance with physical expectations. We examined the Pearson correlation of muscle groups to target knee angles, the results are shown in Figure 7 for all study participants clustered by muscle groups. The quadriceps muscles (Rectus Femoris, Vastus Lateralis, Vastus Medialis) are inversely correlated with the knee angle, while the posterior muscles (Semitendinosus, Biceps Femoris) show weak positive or no correlation as they don't take an active part in the leg extension effort. Tibialis Anterior muscles of the lower leg show a tendency for positive correlation, and no common correlation among participants is prominent for Medial Gastrocnemius.

### 5.2. Simulated Knee Extension

**Figure 8:**
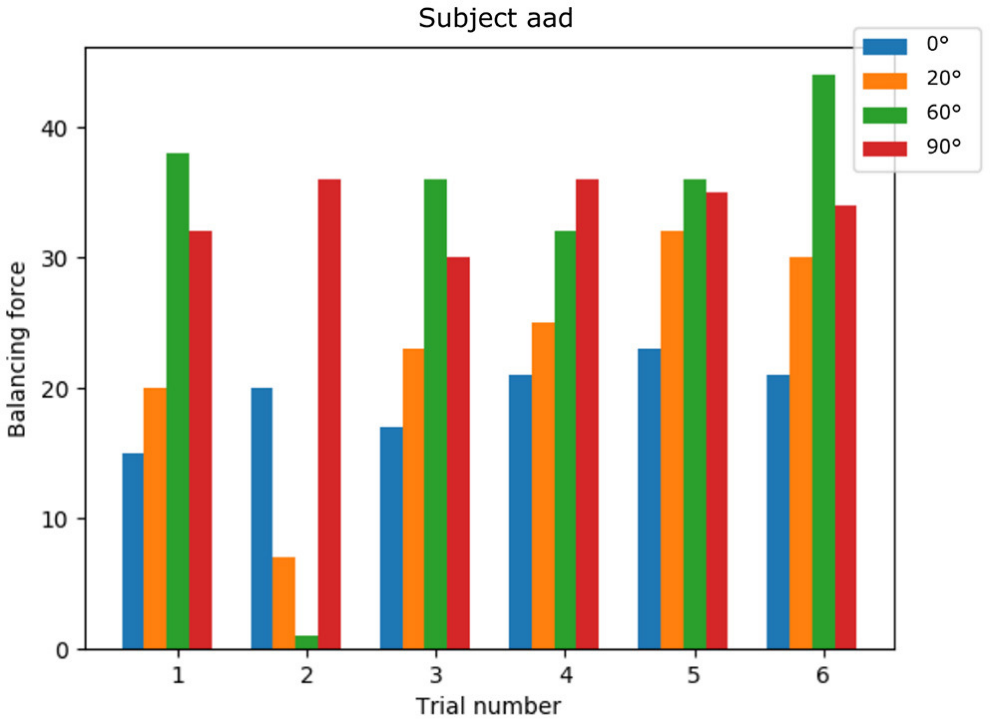
Knee torque for a single subject: generated torques show a common pattern across trials and non-optimal trial executions can be identified.

The NRP has proven crucial in analyzing the resulting knee torque by applying the EMG derived muscle activations. The balancing torque required was different for each subject, which reflects different anatomical configurations. Also the angle at which the maximal force was induced varied, indicating different strategies of executing the knee extension task among participants. Figure 8 shows balancing forces for an exemplary subject with mostly increasing balancing force with increasing knee angle. While difficult to extract from the raw EMG data, using the simulation results we receive a clear picture of which trials the subject might not have performed the maximum voluntary effort study experiment correctly. Here in trial number 2 balancing forces for 20 and 60 degree are significantly lower compared to other trials of the same study participant.

### 5.3. Comparison of EMG Derived and GA Generated Muscle Activation

Our experiments have shown that the GA reaches a conclusion fairly rapidly after around 20 generations. However, since the fitness function waits a couple of seconds for the simulation to reach a stable position with the evaluation of a new individual, the entire process may take up to tens of minutes.

Comparing natural (extracted from the experimental study data) and evolved muscle activations achieving the same extension torque, configurations are not linearly correlated for any given subject and angle. This is to be expected, as even comparing two experiments belonging to two different subjects, recorded at the same angle does not present any relevant similarity. We investigated evolutionary generated muscle activations for all participants, and indeed found very different strategies to provide the given balancing torque. The only consistent characteristic that holds true throughout all subjects for both approaches is the overall dominance of the quadriceps extensor over the posterior contracting muscles. We therefore will present two examples for the corner cases here with each examples from two participants: First, the GA finds a very similar activation pattern as the one extracted from the EMG data, and second the GA finds a very different solution for muscle activation while reaching the same knee torque.

**Figure 9:**
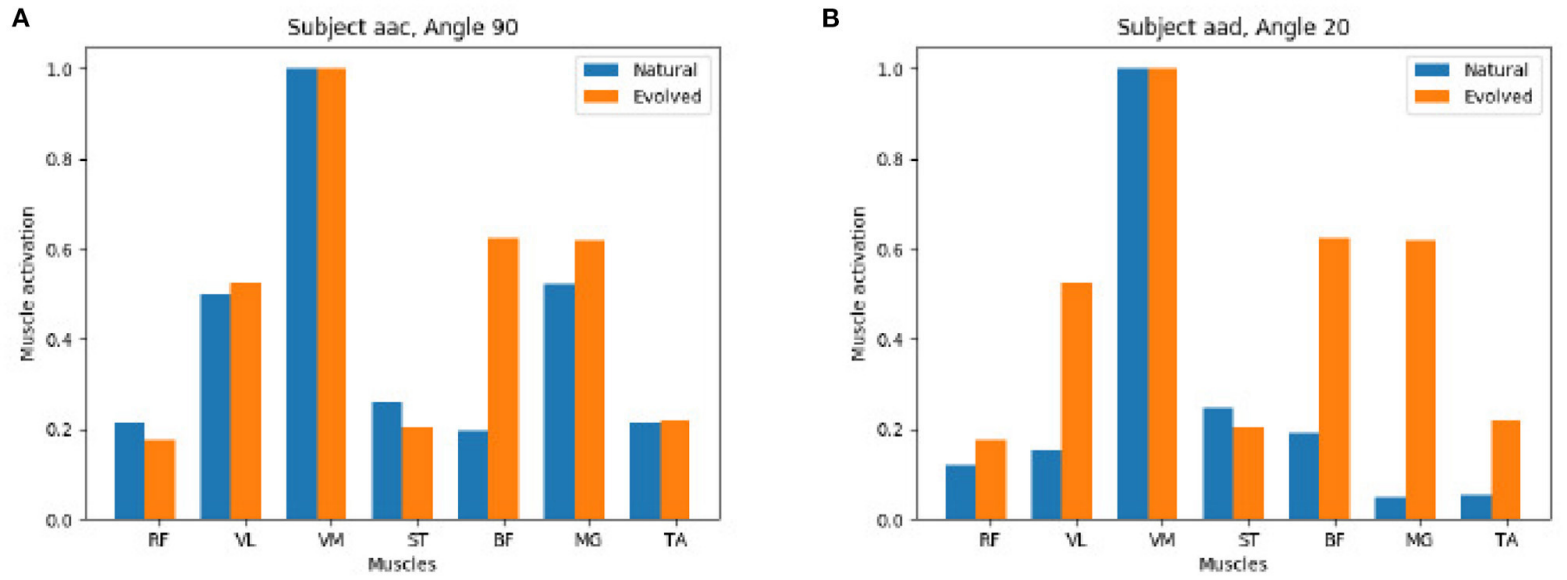
Positive comparison of natural (extracted from the experimental study) vs. evolved (computed by the Genetic Algorithm) synergies: The Genetic Algorithm computes synergies that recreate the muscle activation of processed EMG data of the participant study closely. **(A)** Subject aac Angel 90. **(B)** Subject aad Angel 20.

Figure 9 shows a detailed comparison between the natural and the evolved synergies for two exemplary subjects at a given angle. The GA found a suitable muscle activation pattern to recreate the extension force which closely resembles the original recorded values, in particular we observe the dominance of the VM muscle and on the left we also observe the moderate support of VL and MG muscles in both the natural and evolved muscle activation.

**Figure 10:**
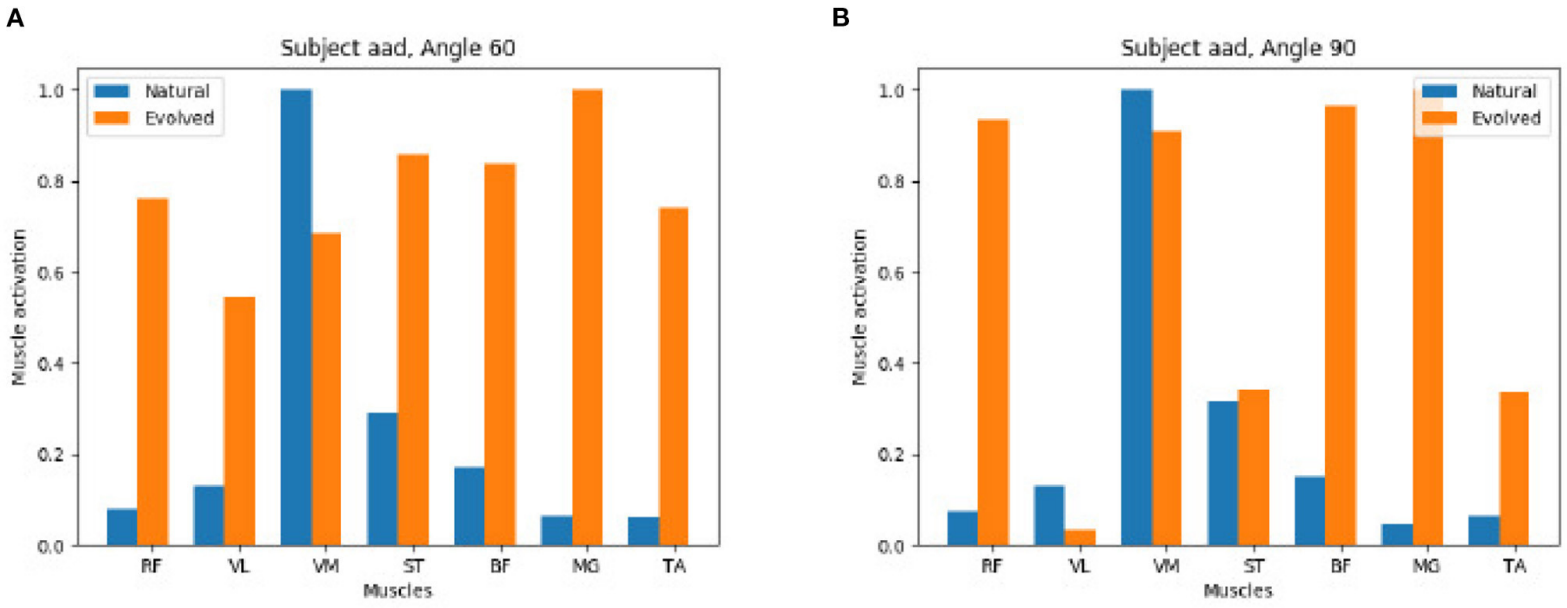
Negative comparison of natural vs. evolved synergies: The Genetic Algorithm recreates the knee torque of study participants but identifies different synergies to reach the same torque. **(A)** Subject aad Angel 60. **(B)** Subject aad Angel 90.

In contrast, Figure 10 shows that the GA is able to reach a valid extension effort end state while its results end up quite distinct from the original, natural configuration. On the left we observe a strong activation of all muscles, on the right a mix of muscles with a strong and weak activation is found.

As a conclusion we observe that the same results, both in pose and in extension torque, can be achieved by different activation patterns.

## 6. Conclusion

In this paper we introduced a simulation framework that uses a virtual musculoskeletal model in the NRP for calculating effective joint torques of recorded surface EMG data. The model skeleton is simulated with rigid body dynamics in Gazebo and extended with hill type muscles of OpenSim. Within our framework we implemented two different control components to actuate the virtual model. First, we analyzed surface EMG data and computed normalized muscle activation to control the simulated model. We could replicate the experimental study virtually and inspected the physical impact of muscle forces and joint torques by applying increasing counteracting torques. Both force values are highly relevant research objectives (Zhou et al., 2011; Zhu et al., 2017) but could only be accessed invasively so far (Roberts and Gabaldón, 2008; Disselhorst-Klug et al., 2009). Furthermore, our implementation makes use of a physics simulated model in a virtual environment and hereby is more generic than other approaches with, e.g., LabView in Zuchruf et al. (2021). Using this model in a second step we implemented a Genetic Algorithm that computes optimal muscle control configurations that can reproduce observed joint torques. Overall, the framework is implemented using ROS interfaces and design tools provided with the NRP and hereby is highly modular and can easily be applied to a variety of musculoskeletal models and experiment scenarios.

In this work, we demonstrated the capabilities of our framework and its advantages for EMG evaluation with specific data from a human knee extension study. For this purpose we integrated a human musculoskeletal model of the lower limbs into the NRP and hereby replicated the physical study setup *in-silico*. In line with the physical constraints applied in the human participant study the simulated leg in the NRP is restricted to motions in a vertical space. However, the simulated model can easily be extended (e.g., in terms of number of muscles, Degrees of Freedom and limb) and hereby opens up the introduced framework for a variety of further studies in the future. Previous work with musculoskeletal models in the NRP focused on rodent motion control paradigms to study stroke rehabilitation in the cortex (Mascaro et al., 2020). We investigated neural activation signals to muscles, the lowest level of neural activation in the motion control hierarchy contributing to a better understanding of the descending pathways of motor control. The data analysis showed that quadriceps muscles are inversely correlated with the knee angle, while the posterior muscles show weak correlations. With the simulated model we demonstrated that even with the same goal knee angles participants applied different muscle configurations to achieve these.

With our GA approach, we can reproduce this variability of synergies from the study even with a generalized simulation model and showcase additional control strategies leading to the same knee torque. The GA suggests solutions that match the experimental outcomes, but also some unlike what the subjects produced—e.g., in individuals there is maximal activity in primary muscles while the GA also finds solutions with increased activity across multiple muscles. This is maybe because GA is unbiased and not working within the human biomechanical constraints.

Overall, the NRP allowed us for the first time to know and reproduce the effective joint torques and muscle synergies that are generated during a task. The observed variability supports the idea that the muscle interactions are not all hardwired or predefined but fairly flexible even if the endpoint or the torque generated is almost the same across all individuals. The observed muscle recruiting requires a neural control system that is less rigid and responsive to the specific needs, as predicted by York et al. (2022) recently, where they show that the proprioceptive inputs are capable of affecting the muscle recruitment pattern depending on the need.

The NRP is designed to foster understanding of neural control paradigms by the *in silico* development of and experimentation with spiking neural networks controlling (musculoskeletal) models interacting in virtual environments. Future work of various research groups will focus on the implementation of such embodied closed-loop computational neural network models in the NRP that mimic neural motion control in the spinal cord. Our introduced pipeline provides a basis to validate resulting muscle activation and biomechanical motion patterns generated by these computational models. To validate such functional models with our proposed methods we provide the muscle activation of the biological ground truth (EMG data) as well as any possible reference solutions (Genetic Algorithm) to achieve the same motion behavior for the given knee extension use case or any other application our method may be transferred to.

## Data Availability Statement

The data analyzed in this study is subject to the following licenses/restrictions: the datasets are available upon request as per the data sharing rules of the University of Leeds. Requests to access these datasets should be directed to SC, S.Chakrabarty@leeds.ac.uk.

## Ethics Statement

The studies involving human participants were reviewed and approved by Local Ethics Committee of the University of Leeds (reference number BIOSCI 16-004). The patients/participants provided their written informed consent to participate in this study.

## Author Contributions

BF conceptualized the experiment design of EMG data evaluation in a virtual simulation environment exploiting the NRP simulation in discussion with MK, wrote the paper with text input from MK and SC for the Section 1 and EMG data collection, as well as CS for the analysis and implementation. PS conducted the participant study and collected the EMG data under supervision of SC and SA. Human experiments were designed by PS and SC. AK supervised the NRP development and provided input for the given use case. CS and BF analyzed the data and implemented the experiment in discussion with SC and MK. All authors contributed to the article and approved the submitted version.

## Funding

This project/research has received funding from the European Union's Horizon 2020 Framework Programme for Research and Innovation under the Specific Grant Agreement No. 785907 (Human Brain Project SGA2) and No. 945539 (Human Brain Project SGA3). PS was funded by the Royal Thai Government Scholarships.

## Conflict of Interest

The authors declare that the research was conducted in the absence of any commercial or financial relationships that could be construed as a potential conflict of interest.

## Publisher's Note

All claims expressed in this article are solely those of the authors and do not necessarily represent those of their affiliated organizations, or those of the publisher, the editors and the reviewers. Any product that may be evaluated in this article, or claim that may be made by its manufacturer, is not guaranteed or endorsed by the publisher.
